# Integration Host Factor Modulates the Expression and Function of T6SS2 in *Vibrio fluvialis*

**DOI:** 10.3389/fmicb.2018.00962

**Published:** 2018-05-15

**Authors:** Jingjing Pan, Meng Zhao, Yuanming Huang, Jing Li, Xiaoshu Liu, Zhihong Ren, Biao Kan, Weili Liang

**Affiliations:** ^1^State Key Laboratory of Infectious Disease Prevention and Control, and National Institute for Communicable Disease Control and Prevention, Chinese Center for Disease Control and Prevention, Beijing, China; ^2^Collaborative Innovation Centre for Diagnosis and Treatment of Infectious Diseases, Hangzhou, China

**Keywords:** type VI secretion system (T6SS), integration host factor, regulation of gene expression, bacterial killing, *Vibrio fluvialis*, electrophoretic mobility shift assay (EMSA)

## Abstract

*Vibrio fluvialis*, an emerging foodborne pathogen of increasing public health concern, contains two distinct gene clusters encoding type VI secretion system (T6SS), the most newly discovered secretion pathway in Gram-negative bacteria. Previously we have shown that one of the two T6SS clusters, namely VflT6SS2, is active and associates with anti-bacterial activity. However, how its activity is regulated is not completely understood. Here, we report that the global regulator integration host factor (IHF) positively modulates the expression and thus the function of VflT6SS2 through co-regulating its major cluster and *tssD*2*-tssI*2 (also known as *hcp-vgrG*) orphan clusters. Specifically, reporter gene activity assay showed that IHF transactivates the major and orphan clusters of VflT6SS2, while deletion of either *ihfA* or *ihfB*, the genes encoding the IHF subunits, decreased their promoter activities and mRNA levels of *tssB*2, *vasH*, and *tssM*2 for the selected major cluster genes and *tssD*2 and *tssI*2 for the selected orphan cluster genes. Subsequently, the direct bindings of IHF to the promoter regions of the major and orphan clusters were confirmed by electrophoretic mobility shift assay (EMSA). Site-directed mutagenesis combined with reporter gene activity assay or EMSA pinpointed the exact binding sites of IHF in the major and orphan cluster promoters, with two sites in the major cluster promoter, consisting with its two observed shifted bands in EMSA. Functional studies showed that the expression and secretion of hemolysin-coregulated protein (Hcp) and the VflT6SS2-mediated antibacterial virulence were severely abrogated in the deletion mutants of Δ*ihfA* and Δ*ihfB*, but restored when their trans-complemented plasmids were introduced, suggesting that IHF mostly contributes to environmental survival of *V*. *fluvialis* by directly binding and modulating the transactivity and function of VflT6SS2.

## Introduction

The type VI secretion system (T6SS) is the most recently discovered contact-dependent protein secretion system in Gram-negative bacteria (Alteri and Mobley, 2016). Although T6SS is encoded within gene clusters that vary in genetic contents and organization in diverse bacteria (Filloux et al., 2008), a minimal set of 13 core T6SS genes have been recognized (Boyer et al., 2009; Silverman et al., 2012). Structurally, T6SS mimics a contractile phage tail in a topologically reversed orientation (Basler et al., 2012; Ho et al., 2014), and functionally, it acts as a virulence determinant against eukaryotic host cells or is involved in interbacterial interactions and competition functions (Pukatzki et al., 2006, 2007; Russell et al., 2014; Alteri and Mobley, 2016).

The T6SS operating is energetically costly to bacterial cells, so its gene cluster is tightly controlled to adapt its expression and assembly to changing environmental conditions (Journet and Cascales, 2016). Environmental cues, such as temperature (Ishikawa et al., 2012; Salomon et al., 2013; Huang et al., 2017), salinity/osolarity (Ishikawa et al., 2012; Huang et al., 2017), iron limitation (Brunet et al., 2011), stresses (Gueguen et al., 2013), cell lysates (LeRoux et al., 2015) etc., affect the expression of T6SS in various species. At present, VasH is the first identified T6SS regulator encoded within the *Vibrio cholerae* T6SS major cluster, which works as a transactivator of T6SS in *V. cholerae* together with σ^54^ (Bernard et al., 2011; Kitaoka et al., 2011). Additional regulators, including ferric-uptake regulator (Fur) and histone-like nucleoid structuring protein (H-NS), were found to repress T6SS in different bacterial strains (Brunet et al., 2011; Chakraborty et al., 2011; Salomon et al., 2014; Alteri and Mobley, 2016). Quorum sensing coordinates T6SS expression by repressing it at low cell density through four small RNAs activated by phosphorylated LuxO while upregulating it at high cell density through HapR (Shao and Bassler, 2014; Joshi et al., 2017). A posttranslational regulatory system termed the threonine phosphorylation controls the T6SS expression in *Pseudomonas aeruginosa* (Mougous et al., 2007). Further defining the activation signals, exploring novel regulators and characterizing the regulatory modules of T6SS are helpful to broaden our understanding of its function in specific bacteria species under the surviving niches and pathogenicity process.

We previously sequenced the whole genome of a clinical isolate of *V. fluvialis*, an emerging foodborne pathogen of increasing public health concern, whose sequence analysis revealed the existence of T6SS homologous gene clusters (Lu et al., 2014). Subsequently, we characterized the organization, function, and expression regulation of T6SS in *V. fluvialis* (Huang et al., 2017). We showed that one of the two T6SSs, termed VflT6SS2, is functionally active under low (25°C) and warm (30°C) temperatures. The functional expression of VflT6SS2 is associated with antibacterial activity which is Hcp-dependent and requires the transcriptional regulator VasH as in *V. cholerae* (Kitaoka et al., 2011; Huang et al., 2017). The genetic composition and organization of VflT6SS2 in *V. fluvialis* are highly homologous to the T6SS in *V. cholerae*, except possessing three *hcp-vgrG* orphan clusters, named *tssD*2_*a*-*tssI*2_*a*, *tssD*2_*b*-*tssI*2_*b* and *tssD*2_*c*-*tssI*2_*c* in *V. fluvialis*. Mutation analysis found that single deletion of *tssD*2_*a*, *tssD*2_*b*, or *tssDI*2_*c* had no influence on Hcp secretion as well as VflT6SS2-dependent killing of *Escherichia coli*, but double deletion of *tssD*2_*a* and *tssD*2_*b* significantly decreased Hcp expression and VflT6SS2’s killing function (Huang et al., 2017). However, the mechanism behind the differential contribution of the three *hcps* to VflT6SS2 function is still unclear.

Using reporter fusion assays, we firstly showed that *tssD*2_*a* has the highest expression level, followed by *tssD*2_*b*, and then *tssD*2_*c*, and all their expressions are positively controlled by the transcriptional regulator VasH as showed in *V. cholerae* (Bernard et al., 2011; Kitaoka et al., 2011). Promoter sequence analysis of *tssD*2-*tssI*2 alleles and the VflT6SS2 major cluster revealed the existences of consensus recognition sequence of integration host factor (IHF), a specific DNA-binding protein that functions in genetic recombination as well as transcriptional and translational controls (Freundlich et al., 1992). IHF is a heterodimeric protein composed of IHFα and IHFβ subunits encoded by unlinked *ihfA* and *ihfB* genes, respectively. Deletion of either *ihfA* or *ihfB* results in a significantly reduced transcription of *tssD*2-*tssI*2 alleles and the major cluster operon. Consistently, the expression and secretion of Hcp and the VflT6SS2-mediated antibacterial virulence were severely decreased in the *ihfA* or *ihfB* mutants, but restored with *ihfA* or *ihfB* overexpression from trans-complemented plasmids. Electrophoretic mobility shift assay (EMSA) demonstrated the direct binding of IHF to the promoters of *tssD*2-*tssI*2 and VflT6SS2 major cluster. In addition, sequence mutation analysis further confirmed that the regulatory effect relies on the binding of IHF to its consensus recognition sites. In summary, in this study, we made clear the differential expression patterns of *tssD*2-*tssI*2 clusters and demonstrated that IHF directly and positively regulates VflT6SS2 expression in *V. fluvialis* by co-transactivating both the *tssD*2-*tssI*2 orphan clusters and the VflT6SS2 major cluster, thus contributing to the survival of bacteria in highly competitive environments.

## Materials and Methods

### Bacterial Strains, Culture Conditions, and Plasmids

The wild-type (WT) *V. fluvialis* 85003 and its derivative mutants were grown in Luria-Bertani (LB) broth (pH7.4) containing 1% NaCl (170 mM) at 30°C unless specifically indicated. *E. coli* DH5α*λpir* and SM10*λpir* were routinely cultured at 37°C and used for cloning purposes. Culture media were supplemented with ampicillin (Amp, 100 μg/ml), streptomycin (Sm, 100 μg/ml), tetracycline (Tc, 10 μg/ml for *E. coli*, 2.5 μg/ml for *V. fluvialis*), chloramphenicol (Cm, 10 μg/ml), rifampicin (Rfp, 50 μg/ml), or isopropyl-β-D-thiogalactopyranoside (IPTG) as required. All strains and plasmids used in this study are listed in **Table 1**.

**Table 1:** Strains and plasmids used in this study.

Strain/plasmid	Characteristics	Reference/source
***E. coli***		
DH5α*λpir*	*sup E44*Δ*lacU169* (Φ*lacZ*Δ*M15*) *recA1 endA1 hsdR17 thi-1 gyrA96 relA1pir* (Laboratory stock)	Mekalanos Laboratory (Harvard Medical School)
SM10λ*pir*	*thi thr leu tonA lacY supE recA*::RP4-2Tc::Mu (λ*pir*R6K), Km^R^	Mekalanos Laboratory (Harvard Medical School)
MG1655	K-12 F^-^ λ^-^ *ilvG*^-^ *rfb-50 rph-1*, Rfp^R^	Laboratory stock
***V. fluvialis***		
85003	*V. fluvialis*, wild-type, Sm^R^	Lu et al., 2014
Δ*ihfA*	85003, Δ*ihfA*	This study
Δ*ihfB*	85003, Δ*ihfB*	This study
Δ*vasH*	85003, Δ*vasH*	Huang et al., 2017
Δ*ihfA*/pSR*ihfA*	Δ*ihfA* containing complemented plasmid pSR*ihfA*	This study
Δ*ihfA*/pSRKTc	Δ*ihfA* containing control vector pSRKTc	This study
Δ*ihfB*/pSR*ihfB*	Δ*ihfB* containing complemented plasmid pSR*ihfB*	This study
Δ*ihfB*/pSRKTc	Δ*ihfB* containing control vector pSRKTc	This study
**Plasmid**		
pWM91	Suicide vector containing R6K ori, *sacB*, *lacZ*α; Amp^R^	Laboratory stock
pSRKTc	Broad-host-range vector containing *lac* promoter, *lacI*^q^, *lacZ*α,Tet^R^	Khan et al., 2008
pBBR*lux*	bioluminescence based reporter plasmid containing a promoterless *luxCDABE* operon; Cm^R^	Wu et al., 2015
pWM-Δ*ihfA*	1.69 kb *BamH*I-*Xho*I Δ*ihfA* fragment of *V. fluvialis* in pWM91	This study
pWM-Δ*ihfB*	1.70 kb *BamH*I-*Xho*I Δ*ihfB* fragment of *V. fluvialis* in pWM91	This study
pSR*ihfA*	313 bp *Nde*I-*Xho*I *ihfA* ORF of *V. fluvialis* in pSRKTc	This study
pSR*ihfB*	304 bp *Nde*I-*Xho*I *ihfB* ORF of *V. fluvialis* in pSRKTc	This study
p*tssD*2*a*-*lux*	375 bp *Sac*I-*BamH*I fragment of *tssD*2_*a* promoter region in pBBR*lux*	This study
p*tssD*2*a*M-*lux*	375 bp *Sac*I-*BamH*I fragment of *tssD*2_*a* promoter with IHF consensus mutation in pBBR*lux*	This study
p*tssD*2*b*-*lux*	375 bp *Sac*I-*Spe*I fragment of *tssD*2_*b* promoter region in pBBR*lux*	This study
p*tssD*2*c*-*lux*	604 bp *Sac*I-*BamH*I fragment of *tssD*2_*c* promoter region in pBBR*lux*	This study
p*tssD*2*c′*-*lux*	395 bp *Sac*I-*BamH*I fragment of shortened *tssD*2_*c* promoter region in pBBR*lux*	This study
pVflT6SS2-*lux*	450 bp *Sac*I-*BamH*I fragment of VflT6SS2 major cluster promoter region in pBBR*lux*	This study
pVflT6SS2-*lux*-*ihf*1M	450 bp *Sac*I-*BamH*I fragment of VflT6SS2 promoter with mutations in the first IHF binding site in pBBR*lux*	This study
pVflT6SS2-*lux*-*ihf*2M	450 bp *Sac*I-*BamH*I fragment of VflT6SS2 promoter with mutations in the second IHF binding site in pBBR*lux*	This study
pVflT6SS2-*lux*-*ihf*3M	450 bp *Sac*I-*BamH*I fragment of VflT6SS2 promoter with mutations in the third IHF binding site in pBBR*lux*	This study
pVflT6SS2-*lux*-*ihf*1+2M	450 bp *Sac*I-*BamH*I fragment of VflT6SS2 promoter with mutations in the first and second IHF binding sites in pBBR*lux*	This study

### Construction of Transcriptional Reporteeporter Plasmids

Promoter regions of *tssD*2_*a*, *tssD*2_*b*, *tssD*2_*c*, and VflT6SS2 major cluster were amplified by polymerase chain reaction (PCR) using the Prime STAR^®^ HS DNA Polymerase (TaKaRa, Dalian, China), and the products were cloned into pBBR*lux*, which contains a promoterless *luxCDABE* reporter. The resultant recombinant constructs were named p*tssD*2*a*-*lux*, p*tssD*2*b*-*lux*, p*tssD*2*c*-*lux*, and pVflT6SS2-*lux.* p*tssD*2*a*M-*lux*, pVflT6SS2-*lux*-*ihf*1M, pVflT6SS2-*lux*-*ihf*2M, pVflT6SS2-*lux*-*ihf*3M, and pVflT6SS2-*lux*-*ihf*1+2M plasmids, which contain single or double site mutations in the predicted IHF binding sites, were generated by PCR-based site-directed mutagenesis using p*tssD*2*a*-*lux*, pVflT6SS2-*lux* or pVflT6SS2-*lux*-*ihf*1M as a template. Truncated p*tssD*2*c*′-*lux* was yielded by overlap extension PCR using p*tssD*2*c*-*lux* as the template. The detailed information about these constructs is listed in **Table 1**, and all the constructs were confirmed by sequencing. Primer sequences used here are shown in **Table 2**.

**Table 2:** Primers used in this study.

Primers	Oligonucleotide sequences (5′-3′)^∗^
pHcp-up-*Sac*I	CCCGAGCTCAGTCCGTCGCCATCAAATAG
pHcp-dn-*BamH*I	CGGGATCCGAGTTTGACCTTCGATAGAG
pHcp-A-up-*Sac*I	CCCGAGCTCTGAGAATAGCCTTCCTTGAC
pHcp-B-up-*Sac*I	CCCGAGCTCGTGCCACCTTTGGCTACGTT
pHcp-B-dn-*Spe*I	GGACTAGTGAGTTTGACCTTCGATAGAG
pHcp-C′-dn	CCAACTGGGCAATAACAAATAATCAATAAGTT AGCGC
pHcp-C′-up	TTGATTATTTGTTATTGCCCAGTTGGCAAGTTAT
pHcp-A-M-up	GGCAAGGTTTTAAATATCACTAATACCTTTT AAAAGAATGCCAAAGTGG
pHcp-A-M-dn	TTTGGCATTCTTTTAAAAGGTATTAGTGATA TTTAAAACCTTGCCATTAGA
pT6SS2-up-*Sac*I	ACGAGCTCACCATGATCTGTTCTGGGAT
pT6SS2-dn-*BamH*I	CGGGATCCTTAGGAGCTACACTTCCTTC
pT6SS2-1M-dn	TCTATTCATTTAATCATGTTTACGTGCACAAA AATCACAAGAATA
pT6SS2-1M-up	TGATTTTTGTGCACGTAAACATGATTAAATGAA TAGAATGTGCTCG
pT6SS2-2M-dn	TTTTTAGTAAATATCACTTCTACCAATTGATTA ATTCACCCGACTT
pT6SS2-2M-up	AATTAATCAATTGGTAGAAGTGATATTTACTAA AAATCAAATAAGATA
pT6SS2-3M-dn	AATAGTTGTGTCAATATCACCTTGATAATACAC ATTAGAAATATC
pT6SS2-3M-up	TGTGTATTATCAAGGTGATATTGACACAACTAT TTCATTGACAAC
vflihfA-F1-up-*BamH*I	CGGGATCCGGAGAGTGAATGAGCCTA
vflihfA-F1-dn	TTTTATGGCGGTCGTAAAAAGACCGAGC
vflihfA-F2-up	TTTTTACGACCGCCATAAAACTTCCCTC
vflihfA-F2-dn-*Xho*I	CCGCTCGAGTCACCCTGAGCTTGAACG
vflihfB-F1-up-*BamH*I	CGGGATCCTGGTTCGTCGACAAGCTG
vflihfB-F1-dn	AAACTATGACCGAAAACATTTGATTTACG
vflihfB-F2-up	AATGTTTTCGGTCATAGTTTCCCTCATCG
vflihfB-F2-dn-*Xho*I	CCGCTCGAGCAGTCATTCGCTGAAGCAC
vflihfA-F-*Xho*I	CCGCTCGAGTTACGACTTTTTAATGTTC
vflihfA-R-*Nde*I	GGAATTCCATATGGCGCTCACAAAGGC
vflihfB-F-*Xho*I	CCGCTCGAGTCAAATGTTTTCGTTTACA
vflihfB-R-*Nde*I	GGAATTCCATATGACTAAGTCTGAATTG
VF-recA-qPCR-up	ACCGAGTCAACGACGATAAC
VF-recA-qPCR-dn	TGATGAACTGCTGGTGTCTC
qvipA/tssB2-F	CTGACGACAACAGTGAAGAAC
qvipA/tssB2-R	TGCGAAGCCACAGAATCC
hcp-qPCR-F-com	TCGGCGATTCATTCGTT
hcp-qPCR-R-com	CAGTTCAACCGTCGTCATCT
vgrG-qPCR-F-AB	GCATCTTCCAACTCAACAC
vgrG-qPCR-R-AB	GTACACCAGCCCTTCTTC
VF-vasK-qPCR-F	ACATCCAACGCCAATACG
VF-vasK-qPCR-R	CAATCGCAGTGAAGACAAC
VF-vasH-qPCR-F	GGTAATCGGATACTGGAAC
VF-vasH-qPCR-R	CATGTCAACTTGCTGGAT
HcpA-up-Biotin	TGAGAATAGCCTTCCTTGAC
HcpA-dn-Biotin	GAGTTTGACCTTCGATAGAG
T6SS2-up-Biotin	ACCATGATCTGTTCTGGGAT
T6SS2-dn-Biotin	TTAGGAGCTACACTTCCTTC

*^∗^The underlined bases indicate the restriction enzyme sites.*

### Construction of Mutants and Complementation Plasmids

In-frame deletion mutants Δ*ihfA* and Δ*ihfB* were constructed by allelic exchange using 85003 as a precursor. Briefly, chromosomal DNAs flanking the *ihfA* and *ihfB* open reading frames (ORFs) were amplified with primer pairs listed in **Table 2**. The amplified upstream and downstream DNAs of the target genes were stitched together by overlapping PCR as described previously (Wu et al., 2015). The resulting 1.69 kb Δ*ihfA* and 1.70 kb Δ*ihfB* fragments were cloned at *BamH*I/*Xho*I sites into pWM91 suicide plasmid. The resultant recombinant plasmids, pWM-Δ*ihfA* and pWM-Δ*ihfB*, were mobilized into the strain 85003 from *E. coli* SM10*λpir* by conjugation. Exconjugants were selected in LB medium containing Amp and Sm and counter-selected by growing on LB agar containing 15% sucrose. Sucrose-resistant colonies were tested for Amp sensitivity, and mutant allele was verified by PCR and further confirmed by DNA sequencing. The construction procedure for *vasH* mutant was described previously (Huang et al., 2017).

Complementation plasmids, pSR*ihfA* and pSR*ihfB*, were constructed by cloning the *ihfA* and *ihfB* coding sequences into pSRKTc using *Nde*I/*Xho*I sites. The *ihfA* and *ihfB* were expressed from the *lac* promoter with the induction of IPTG.

### Luminescence Activity Assay

*Vibrio fluvialis* strain containing *lux* reporter fusion plasmids was grown overnight with shaking, diluted 1:100 in fresh LB, and 200 μl aliquots were transferred into an opaque-wall 96-well microtiter plate (Ostar 3917). The plates were incubated at 30°C or 37°C with agitation. The optical density at 600 nm (OD_600_) and luminescence were measured by using a microplate reader (Infinite M200 Pro, Tecan). Luminescence activity is calculated as light units/OD_600_ after the light units and OD_600_ were blank-corrected.

### Quantitative Reverse Transcription PCR (qRT-PCR)

*Vibrio fluvialis* strains were grown in LB medium to OD_600_ 1.5. Total RNA extraction and cDNA synthesis were performed as described previously (Wu et al., 2015). qRT-PCR was performed by CFX96 (Bio-Rad) using SYBR Premix Ex Taq (TaKaRa, Dalian, China). Relative expression values (*R*) were calculated using the equation *R* = 2^-(ΔCqtarget-ΔCqreference)^, where Cq is the fractional threshold cycle. The *recA* mRNA was used as an internal reference. A control mixture using total RNA as a template was performed for each reaction to exclude chromosomal DNA contamination. The primers used for these target genes, *recA*, *tssD*2 (*hcp*), *tssI*2 (vgrG), *tssB*2 (*vipA*), *vasH*, and *tssM*2 (*vasK*), were listed in **Table 2**.

### Analyses of VflT6SS2 Expression and Secretion

Overnight cultures of *V. fluvialis* were diluted 1:100 in 5 mL fresh LB and incubated to OD_600_ of 1.5 with shaking at 30°C. In complementation assays, Δ*ihfA/*pSR*ihfA* or Δ*ihfB/*pSR*ihfB* were grown to OD_600_ of 0.5 with Tc. Then, each culture was divided in half. One half was induced by the addition of IPTG (final concentration of 0.5 mM), and the other half was used as a control. The cultures were continually incubated for 3 h with shaking. Δ*ihfA* and Δ*ihfB* containing pSRKTc were used as controls. Protein samples from cell pellets and cell-free supernatants were prepared as previously described with minor modifications (Huang et al., 2017). Trichloroacetic acid precipitated proteins from 1 ml cell-free culture supernatant were suspended in 100 μl RIPA lysis buffer (mild) (ComWin Biotech, Beijing, China). Cell pellets from 1 ml culture were suspended in 200 μl RIPA lysis buffer (mild). After 30 min incubation on ice, samples were centrifuged at 13000 rpm for 30 min at 4°C and supernatants were normalized to the amount of total protein as assayed by the BCA^TM^ protein assay (Thermo Fisher Scientific, United States). Western blot analysis was performed as described previously using polyclonal rabbit anti-Hcp antibody and anti-*E. coli* cyclic AMP receptor protein (CRP) antibody (BioLegend, United States) (Huang et al., 2017).

### Bacterial Killing Assay

Bacterial killing assay was used to evaluate the antibacterial virulence of *V. fluvialis* and performed as described previously with *E. coli* MG1655 as the prey strain (Huang et al., 2017). *V. fluvialis* predator strains 85003, Δ*ihfA* and Δ*ihfB* were grown overnight on LB agar containing 2% NaCl (340 mM) at 30°C. For complementation strains, Δ*ihfA*/pSR*ihfA* and Δ*ihfB*/pSR*ihfB*, a 2-h extra induction with IPTG in LB was included to fully induce *ihfA* and *ihfB* expressions. The colony-forming units (CFU) per milliliter of the prey *E. coli* at the beginning (0 h) and after 5-h incubation with predator (5 h) were determined by plating 10-fold serial dilutions on Sm and Rfp resistant agar plates. Strain with control vector was used as a negative control.

### Electrophoretic Mobility Shift Assay (EMSA)

The 375 bp probes for the wild-type and mutated *tssD*2*a* promoter regions were amplified with primer pair HcpA-up-Biotin/HcpA-dn-Biotin using plasmids p*tssD*2*a*-*lux* and p*tssD*2*a*M-*lux* as templates, respectively. The 450 bp probe for the VflT6SS2 major cluster promoter was amplified with primer pair T6SS2-up-Biotin/T6SS2-dn-Biotin using pVflT6SS2-*lux* as a template. Binding reactions were performed by mixing 20 ng biotin-labeled probe with increasing amounts of purified *V. cholerae* IHF heterodimers in a volume of 20 μl containing binding buffer [50 mM Tris–HCl (pH 8.0), 100 mM KCl, 1 mM dithiothreitol, 2.5 mM EDTA, 5% glycerol], 0.5 μg of calf thymus DNA, and 5 μg/ml bovine serum albumin. The reaction mixture was incubated at room temperature for 30 min, and then separated on a 6% native polyacrylamide gel. The separated free probe DNA and DNA-protein complexes were transferred onto nylon membranes and visualized with the Chemiluminescent Nucleic Acid Detection Module (Thermo Fisher Scientific, United States) following the manufacturer’s instructions. The above primer sequences were displayed in **Table 2**. The constructions of the plasmids expressing *V. cholerae* IHFα and IHFβ subunits and their expressions and purifications will be introduced elsewhere (Li et al., manuscript in preparation).

## Results

### Expressions of Three *tssD*2-*tssI*2 Clusters in *V. fluvialis* VflT6SS2

**FIGURE 1:**
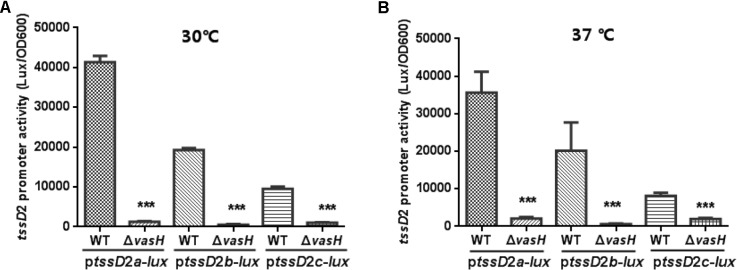
Promoter activities of p*tssD*2*a*-*lux*, p*tssD*2*b*-*lux* and p*tssD*2*c*-*lux* under different culture temperatures. Overnight cultures of the *V. fluvialis* strains 85003 (WT) or Δ*vasH* containing either p*tssD*2a-*lux*, p*tssD*2b-*lux*, or p*tssD*2c-*lux* reporter fusions were diluted 1:100 in LB medium and 200 μl aliquots were transferred to Opaque-wall 96-well microtiter plates. The plates were incubated at 30°C **(A)** or 37°C **(B)** with shaking for measuring the OD_600_ and light units. Luminescence activity is calculated as light units/OD_600_. The data represent three independent experiments. ^∗∗∗^Significantly different between WT and Δ*vasH* mutant (*t*-test, *P <* 0.001).

Our previous study showed that *V. fluvialis* VflT6SS2 contains three *tssD*2-*tssI*2 alleles on different chromosomal locations which are involved in interbacterial competition and the anti-bacterial activity requires transcriptional regulator VasH (Huang et al., 2017). To further dissect the expression and contribution of each allele, we constructed p*tssD*2*a*-*lux*, p*tssD*2*b*-*lux*, and p*tssD*2*c*-*lux* reporter plasmids and introduced them into WT and isogeneic Δ*vasH* mutant and measured the heterogeneous promoter-driven luminance activity at 30°C culture conditions. As shown in **Figure 1A**, in the WT, p*tssD*2*c*-*lux* has the lowest promoter activity which is only one fourth of that of p*tssD*2*a*-*lux*, the one with the highest activity. The activity of p*tssD*2*b*-*lux* falls in between. In the *vasH* deletion background, the promoter activities of the three alleles are all very low compared to the WT. These results are consistent with our previous *tssD*2 mutations’ phenotypes (Huang et al., 2017) and provide a possible explanation why expression of *tssD*2_*c* alone cannot maintain the function of VflT6SS2 in terms of Hcp secretion and interbacterial virulence. Our results also show that although three *tssD*2-*tssI*2 alleles in *V. fluvialis* have differential expression profiles, they are all positively regulated by VasH as in *V. cholerae* (Dong and Mekalanos, 2012).

The VflT6SS2 was previously shown to be unfunctional at 37°C with extremely low *tssD*2 mRNA levels (Huang et al., 2017), so we wondered whether this is due to very low transcription of the three *tssD2* alleles under this temperature. So we measured the promoter activities of p*tssD*2*a*-*lux*, p*tssD*2*b*-*lux*, and p*tssD*2*c*-*lux* at 37°C culture condition. Beyond our expectation, the transcription levels of p*tssD*2*a*-*lux*, p*tssD*2*b*-*lux*, and p*tssD*2*c*-*lux* at 37°C are nearly comparable to that at 30°C (**Figure 1B**), suggesting a post-transcriptional regulation is probably involved in the rapid degradation of *hcp* (*tssD*2) transcripts.

### Bioinformatics Analysis of the Promoter Regions of the Three *tssD*2-*tssI*2 Clusters in *V. fluvialis*

**FIGURE 2:**
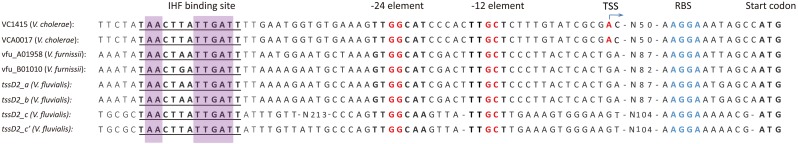
Characteristics of the promoters of *hcp* homologs among different *Vibrio* species. The *hcp* promoter sequences from *V. cholerae*, *V. furnissii*, and *V. fluvialis* were compared which share highly sequence homology in T6SS. The IHF binding sites are underlined, and the conserved bases in *E. coli* are indicated with purple background. The potential ribosome binding sites (RBS) are labeled with blue bases. The –24 and –12 elements of σ^54^ binding sites are marked with red bases. The transcriptional start sites (TSSs) of VC1415 (*hcp*-1) and VCA0017 (*hcp*-2) are designated with an arrow according to that reported in a serotype O17 *V. cholerae* strain (Williams et al., 1996).

To gain insight into the regulation of *tssD*2**-***tssI*2 alleles, we first inspected the sequence features of the promoter regions of *tssD*2_*a*, *tssD*2_*b* and *tssD*2_*c*. σ^54^ (-12/-24) consensus sequences and putative IHF binding site were predicted in all three alleles’ promoters (**Figure 2**). The presence of σ^54^ consensus sequences indicates the dependence of Eσ^54^ for the transcription of the *tssD*2 clusters, which is in agreement with the requirement of VasH for the promoter activities (**Figure 1**). VasH functions as a specialized activator which binds to σ^54^ and induces conformational rearrangement in the Eσ^54^ closed complex (Kitaoka et al., 2011). In addition, a 13-bp asymmetric consensus sequence T**AA**CTTA**TTGAT**T within the three promoters was identified which excellently matches with *E. coli* IHF consensus sequence Y**AA**NNNN**TTGAT**W, where Y stands for T or C, N for any base, and W for A or T (Craig and Nash, 1984). Moreover, the σ^54^ consensus sequences and IHF binding sites show similar sequence intervals among *hcp* homologs from *V. cholerae*, *Vibrio furnissii*, and *V. fluvialis* except for the *tssD*2_*c* which shows a 225-bp instead of 16-bp interval (**Figure 2**).

Considering that the promoter of *tssD*2_*c* displayed the lowest transcription activity in WT compared to those of *tssD*2_*a* and *tssD*2_*b* (**Figure 1**), we wondered whether the 225-bp interval is responsible for its reduced transcription. To test this possibility, we constructed a new reporter fusion, p*tssD*2*c′*-*lux*, which contains a modified *tssD*2_*c* promoter with only 16-bp space between the IHF binding site and the σ^54^ motif (**Figure 2**). However, the p*tssD2c′*-*lux* produced more than sixfold less luminance activity than its WT (data not shown), indicating that the 225-bp sequence likely contains unknown *cis*-acting element(s) essential for its promoter activity and the long sequence spacing is probably not of the reason for the low transcriptional activity of p*tssD*2*c*-*lux*. The underlying mechanism remains to be investigated.

### IHF Positively Regulates *V. fluvialis* VflT6SS2

**FIGURE 3:**
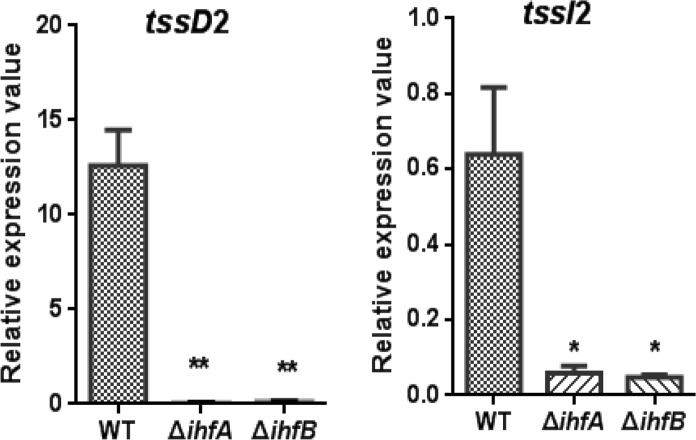
qRT-PCR analysis of the mRNA abundance of *hcp-vgrG* orphan clusters in *V. fluvialis* WT and IHF deletion mutant. *V. fluvialis* 85003 (WT), Δ*ihfA*, or Δ*ihfB* mutant was grown at 30°C in LB medium to around OD_600_ 1.5. RNA was extracted, and the mRNA abundances of *tssD*2 (*hcp*) and *tssI*2 (*vgrG*) were determined by qRT-PCR as described in the Section “Materials and Methods.” The data represent three independent cultures. ^∗∗^Significantly different from WT (*t*-test, *P* < 0.01). ^∗^Significantly different from WT (*t*-test, *P* < 0.05).

**FIGURE 4:**
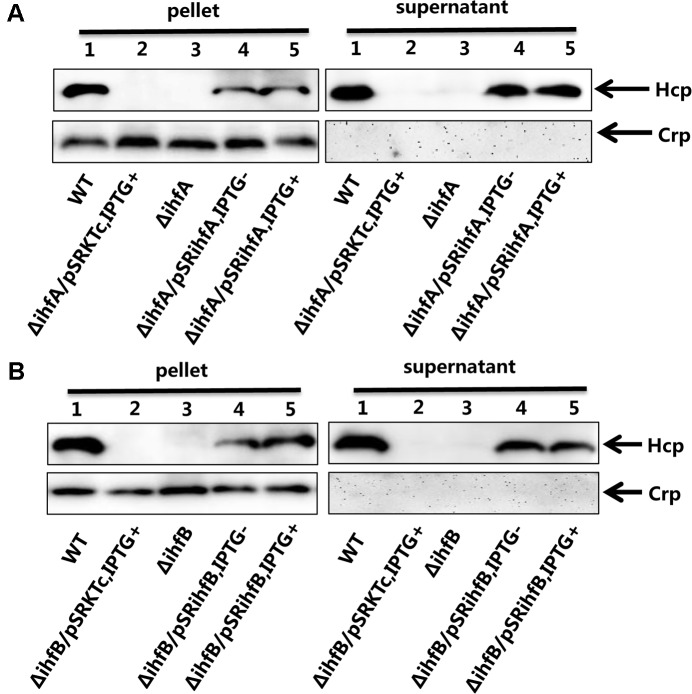
Influence of IHF on the VflT6SS2 Hcp expression and secretion. **(A)** *V. fluvialis* 85003 (WT), Δ*ihfA* mutant, Δ*ihfA* with the empty vector pSRKTc, or with IHFα expression vector pSR*ihfA*. **(B)** *V. fluvialis* 85003 (WT), Δ*ihfB* mutant, Δ*ihfB* with the empty vector pSRKTc, or with IHFβ expression vector pSR*ihfB*. Strains were grown at 30°C in LB medium to around OD_600_ 1.5. Western blot analysis with the anti-Hcp or anti-CRP antibody was performed with 7 μg of total protein extract from the cell pellets and culture supernatants. Lane 1, WT; Lane 2, Δ*ihfA* or Δ*ihfB* with the empty vector pSRKTc with IPTG induction; Lane 3, Δ*ihfA* or Δ*ihfB*; Lane 4, Δ*ihfA* or Δ*ihfB* with corresponding expression vector pSR*ihfA* or pSR*ihfB* without IPTG induction; Lane 5, Δ*ihfA* or Δ*ihfB* with corresponding expression vector pSR*ihfA* or pSR*ihfB* with IPTG induction. The arrows show the immunoblot band to Hcp or Crp. The Crp protein is absent in the culture supernatants, indicating the detection of Hcp in the supernatants was not a consequence of cell lysis.

The IHF is a heterodimeric protein consisting of two subunits, IHFα and IHFβ, encoded by the *ihfA* and *ihfB* genes, respectively. The IHFα (11.0 kDa) and IHFβ (10.8 kDa) subunits in *V. fluvialis* have 45% sequence identity to each other. To assess the regulatory role of IHF on VflT6SS2, we generated Δ*ihfA* and Δ*ihfB* mutants based on strain 85003. We first compared the mRNA levels of *tssD*2 and *tssI*2 between the WT and the *ihf* mutants. As shown in **Figure 3**, the mRNA levels of *tssD*2 and *tssI*2 are significantly reduced in Δ*ihfA* and Δ*ihfB* mutants relative to their WT. Consistently, the expression and secretion of Hcp are completely abolished in these mutants (**Figures 4A,B**, lanes 3), indicating that IHF plays a role in the positive regulation of VflT6SS2. Furthermore, introduction of a complemented plasmid pSR*ihfA* or pSR*ihfB* into corresponding Δ*ihfA* or Δ*ihfB* mutant restored Hcp expression and secretion while introduction of their control vector pSRKTc failed to do so (**Figures 4A,B**, compare lane 2 to lanes 4 and 5). Recovery of Hcp production even occurred in conditions without IPTG induction (**Figures 4A,B**, compare lane 4 to lane 5). These results suggested that IHF is required for expression of *hcp*. The identical phenotypes of the Δ*ihfA* and Δ*ihfB* mutants also imply that IHFα and IHFβ form a complex to modulate Hcp expression in *V. fluvialis*, though differential effects on transcription by deletion of *ihfA* or *ihfB* were reported during culture in rich LB medium (Mangan et al., 2006).

**FIGURE 5:**
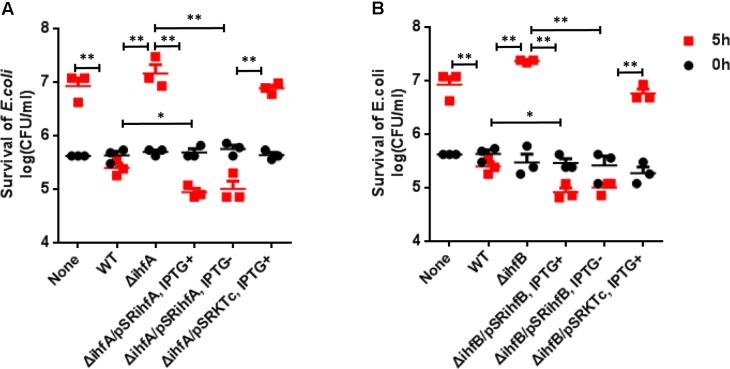
Influence of IHF on the VflT6SS2-dependent competition between *V. fluvialis* and *E. coli* strain MG1655. **(A)** *V. fluvialis* 85003 (WT), Δ*ihfA* mutant, Δ*ihfA* with expression vector pSR*ihfA* or with the empty vector pSRKTc. **(B)** *V. fluvialis* 85003 (WT), Δ*ihfB* mutant, Δ*ihfB* with expression vector pSR*ihfB* or with the empty vector pSRKTc. Bacterial killing assay was performed as described in the Section “Materials and Methods.” The CFU of the prey *E. coli* strain MG1655 was determined at the start point (0 h) and after 5-h (5 h) co-culture with *V. fluvialis* predator at 30°C on LB agar containing 2% NaCl (340 mM). The data represent three independent experiments. None = medium only. WT = wild-type. ^∗∗^Significant differences between sample groups at 5 h (*t*-test, *P <* 0.01). ^∗^Significant differences between sample groups at 5 h (*t*-test, *P <* 0.05).

Previously we have shown that VflT6SS2 plays a role in interbacterial virulence of *V. fluvialis* (Huang et al., 2017). Since IHF regulates the expression of VflT6SS2, we speculate that IHF modulates interbacterial competition through targeting VflT6SS2. Therefore, we performed bacterial killing assay by employing *E. coli* MG1655 as a prey. Our results showed that the colony-forming ability of the *E. coli* prey was retained when co-cultured with Δ*ihfA* (**Figure 5A**) or Δ*ihfB* (**Figure 5B**) mutants, but not with its WT. However, this ability was compromised when incubated with *trans*-complemented strains Δ*ihfA*/pSR*ihfA* and Δ*ihfB*/pSR*ihfB*, regardless of whether *ihfA* or *ihfB* was induced by IPTG or not (**Figures 5A,B**). Strains Δ*ihfA*/pSRKTc and Δ*ihfB*/pSRKTc induced with IPTG were used as controls and showed similar phenotypes to Δ*ihfA* and Δ*ihfB* mutants. Furthermore, under induced condition, the survival of MG1655 incubated with Δ*ihfA*/pSR*ihfA* or Δ*ihfB*/pSR*ihfB* was even lower than that incubated with its WT, which possesses only one copy of *ihfA* and *ihfB* on the chromosome. All together, these results indicate that IHF contributes to the competitive fitness of *V. fluvialis* through activating the VflT6SS2-mediated bactericidal activity.

### IHF Transcriptionally Activates the Expression of *tssD*2-*tssI*2

**FIGURE 6:**
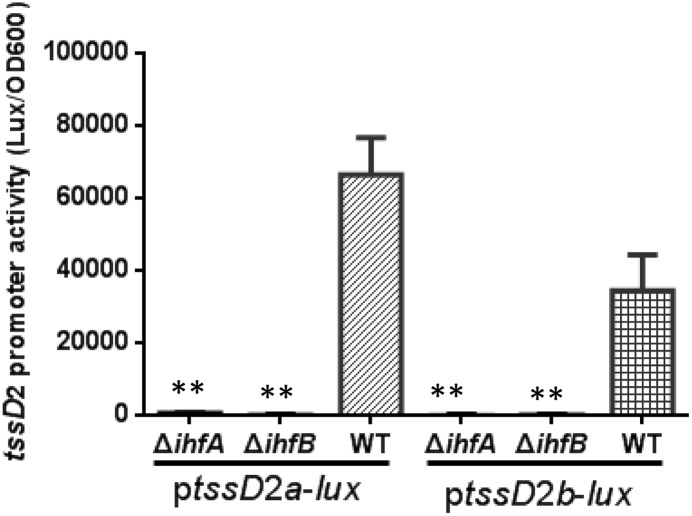
Influence of IHF on *tssD*2*a*-*lux* and *tssD*2*b*-*lux* expression. Overnight cultures of the *V. fluvialis* strains 85003 (WT), Δ*ihfA* or Δ*ihfB* each containing either p*tssD*2a-lux or p*tssD*2b-lux reporter fusions were diluted 1:100 in LB medium and 200 μl aliquots were transferred to Opaque-wall 96-well microtiter plates. The plates were incubated at 30°C with shaking for measuring the OD_600_ and light units. Luminescence activity is calculated as light units/OD_600_. The data represent three independent experiments. ^∗∗^Significantly different from the WT (*t*-test, *P <* 0.05).

The IHF is a sequence-specific DNA-binding protein, and its regulatory function relies on its ability to bend the DNA to which it binds (Robertson and Nash, 1988; Stonehouse et al., 2008; Prieto et al., 2012). The presence of IHF binding sites on *tssD*2 promoters implies a direct transcriptional regulation by IHF. Since *tssD*2_*a* and *tssD*2_*b* are two highly expressed alleles, we focused on these two. First, we introduced the p*tssD*2*a*-*lux* or p*tssD*2*b*-*lux* reporter fusion into WT, Δ*ihfA*, or Δ*ihfB* mutant, and their promoter activities were measured. As shown in **Figure 6**, the luminescence activities of p*tssD*2*a*-*lux* or p*tssD*2*b*-*lux* in Δ*ihfA* and Δ*ihfB* mutants were almost undetectable compared to those in WT, indicating that the promoters of *tssD*2_*a* and *tssD*2_*b* cannot initiate transcription without expression of IHF.

**FIGURE 7:**
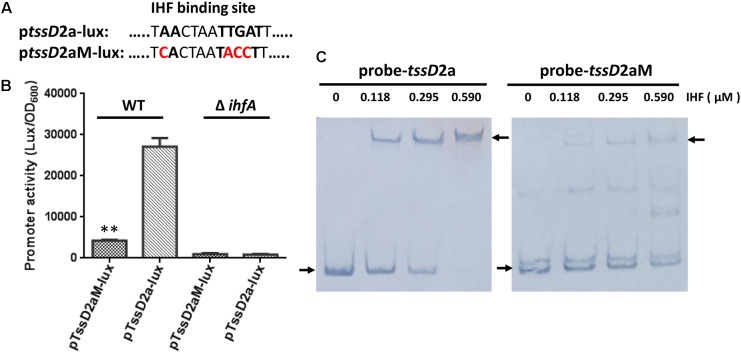
Influence of IHF consensus site mutations on *tssD*2*a* promoter activity and IHF binding. **(A)** The p*tssD*2*a*M-*lux* was constructed by introducing 4-bp changes in the IHF consensus site at the *tssD*2*a* promoter region. **(B)** Overnight cultures of *V. fluvialis* WT and Δ*ihfA* strains containing either p*tssD*2a-*lux* or p*tssD*2*a*M-*lux* reporter fusions were diluted 1:100 in LB medium and 200 μl aliquots were transferred to Opaque-wall 96-well microtiter plates. The plates were incubated at 30°C with shaking for measuring the OD_600_ and light units. Luminescence activity is calculated as light units/OD_600_. The data represent three independent experiments. ^∗∗^Significantly different between the p*tssD*2a-*lux* and the p*tssD*2*a*M-*lux* reporter fusions (*t*-test, *P <* 0.01). **(C)** EMSAs for IHF binding to wild-type *tssD*2_a promoter (left, probe-*tssD*2a) or to its mutations (right, probe-*tssD*2aM). Assays were performed as described in the Section “Materials and Methods.” The biotin-labeled 375-bp DNA probes (20 ng) was incubated with increasing amounts of purified *V. cholerae* IHF protein. The arrow on the left side indicates the unbound free probe, whereas the arrow on the right side indicates the probe bound to IHF protein.

To further demonstrate that IHF relies on the predicted binding site and induces the transcription of *tssD*2, we set out to introduce mutations in the IHF binding site in its promoter region. As the predicted IHF binding sites in *tssD*2_*a* and *tssD*2_*b* are identical (**Figure 2**), we selected *tssD*2_*a* as a representative. We first introduced 4-bp changes in the most highly conserved IHF binding sites by replacing the first A and the TGA with C and ACC, respectively, and named this construct as p*tssD*2*a*M-*lux*(**Figure 7A**). In *V. cholerae*, these mutations have been demonstrated to abolish the binding of IHF to *tcpA* promoter (Stonehouse et al., 2008). Therefore, we compared the promoter activity between p*tssD*2*a*-*lux and* p*tssD*2*a*M-*lux* in WT and Δ*ihfA* mutant. As depicted in **Figure 7B**, the luminescence activity of the p*tssD*2*a*M-*lux* was sixfold less than that of p*tssD*2*a*-*lux* in WT background, but no significant difference was observed in Δ*ihfA* mutant (**Figure 7B**), suggesting that the predicted IHF binding site is required for its effect on transactivation of *tssD*2.

The EMSAs were used to assess whether IHF directly binds to *tssD*2_*a* promoter region. Due to high sequence identity of IHF between *V. cholerae* and *V. fluvialis* (92% for IHFα, 95% for IHFβ), we used purified *V. cholerae* IHF protein in the EMSAs. Increasing amounts of *V. cholerae* IHF were incubated with 20 ng of *tssD*2_a native or mutation-possessing promoter fragments. As shown in **Figure 7C**, IHF did bind *tssD*2_a native promoter. The intensities of the retarded-bands increased in an IHF protein dose-dependent manner and the wild-type promoter fragment was completely shifted in the presence of 590 nM IHF, while the fragment containing the IHF binding mutations failed to efficiently bind IHF. Together, our current findings supported a direct binding of IHF on *tssD*2_a promoter.

### IHF Regulates the Major Cluster of VflT6SS2

**FIGURE 8:**
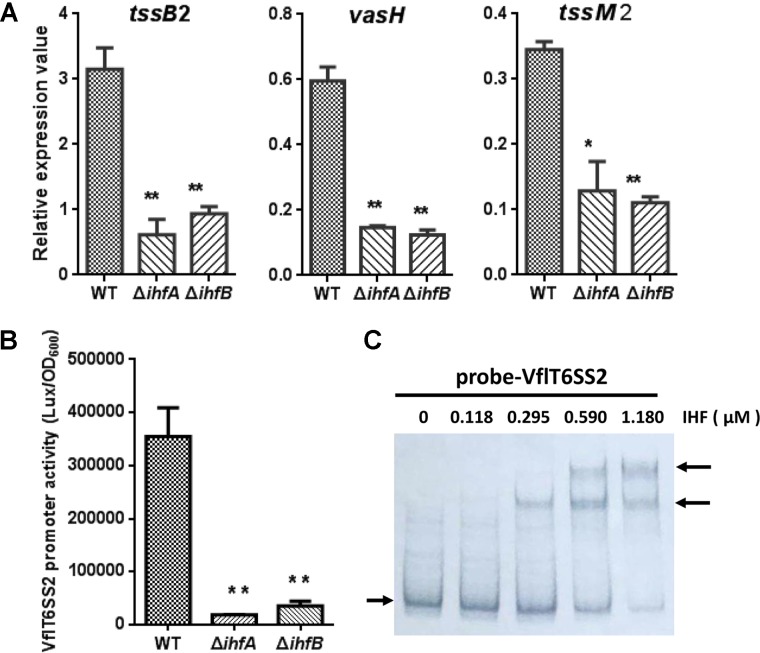
The regulation of IHF on VflT6SS2 major cluster. **(A)** The mRNA abundances of VflT6SS2 major cluster genes *tssB*2, *vasH*, and *tssM*2 in WT and IHF deletion mutants. *V. fluvialis* strains 85003 (WT), Δ*ihfA*, or Δ*ihfB* mutant was grown at 30°C in LB medium to around OD_600_ 1.5. RNA was extracted, and the mRNA abundances of *tssB*2, *vasH*, and *tssM*2 were determined by qRT-PCR. The data represent three independent cultures. ^∗∗^Significantly different from WT (*t*-test, *P* < 0.01). ^∗^Significantly different from WT (*t*-test, *P* < 0.05). **(B)** The transcriptional activity of the VflT6SS2 major cluster promoter in *V. fluvialis* WT and IHF deletion mutants. Overnight cultures of *V. fluvialis* strains 85003 (WT), Δ*ihfA*, or Δ*ihfB* containing the pVflT6SS2-*lux* reporter plasmid were diluted 1:100 in LB medium and 200 μl aliquots were transferred to Opaque-wall 96-well microtiter plate which was incubated at 30°C with shaking for the measurement of the OD_600_ and light units. Luminescence activity is calculated as light units/OD_600_. The data represent three independent experiments. ^∗∗^Significantly different from WT (*t*-test, *P <* 0.01). **(C)** EMSA for IHF binding to the promoter of VflT6SS2 major cluster. Assay was performed as described in the Section “Materials and Methods.” The biotin-labeled 450-bp DNA probes (20 ng) was incubated with increasing amounts of purified *V. cholerae* IHF protein. The arrow on the left side indicates the unbound free probe, whereas the arrow on the right side indicates the probes bound with IHF protein.

The T6SS major cluster and *hcp-vgrG* orphan cluster could be co-regulated or separately controlled by specific regulators. In *V. cholerae*, VasH was shown to regulate two *hcp* operons but not T6SS core cluster (Dong and Mekalanos, 2012). We wondered whether the major cluster of VflT6SS2 is also regulated by IHF. Firstly we scanned the upstream intergenic sequence of *tssB*2 (*vipA*), the first gene of the major cluster, for the putative IHF binding site(s) using the software virtual footprint^1^. This analysis returned three medium-scoring binding sites of IHF. The first putative IHF binding sequence (5′-C**A**C**CAA**AACA**TT**A-3′) is located at nucleotides -297 to -281 relative to *tssB*2 start codon. The second (5′-TT**TCAA**GAAG**TT**A-3′) and the third (5′-A**ATCA**GATAT**TT**A-3′) lie at nucleotides -139 to -124 and -111 to -90, respectively. Generally, these sites are less-conserved and each of them has one mismatch from the *E. coli* IHF consensus sequence (5′-W**ATCAA**NNNN**TT**R-3′, where W = A or T, N = any base, and R = A or G) at different positions (Craig and Nash, 1984). To determine the actual effect of IHF on the transcription of the major cluster of VflT6SS2, we measured the mRNA levels of three selected genes (*tssB*2, *vasH*, and *tssM*2) within the major cluster in WT and IHF deletion mutants. As shown in **Figure 8A**, the abundances of *tssB*2, *vasH*, and *tssM*2 were significantly decreased in Δ*ihfA* and Δ*ihfB* mutants compared to its WT. Then, we constructed the VflT6SS2 major cluster promoter transcriptional fusion, namely pVflT6SS2-*lux*, which was introduced into the WT, Δ*ihfA* or Δ*ihfB* mutant. As expected, the luminescence activities of pVflT6SS2-*lux* were significantly lower in the Δ*ihfA* and Δ*ihfB* mutants compared to the WT, indicating that IHF upregulates the promoter activity of the major cluster of VflT6SS2 to induce its expression (**Figure 8B**). To confirm the direct binding of IHF to the promoter region of the VflT6SS2 major cluster, we performed EMSA. As displayed in **Figure 8C**, IHF was capable of binding VflT6SS2 promoter and two shifted bands appeared with the increase of IHF protein content, suggesting that IHF possibly has two binding sites in the promoter region of the VflT6SS2 major cluster (**Figure 8C**).

**FIGURE 9:**
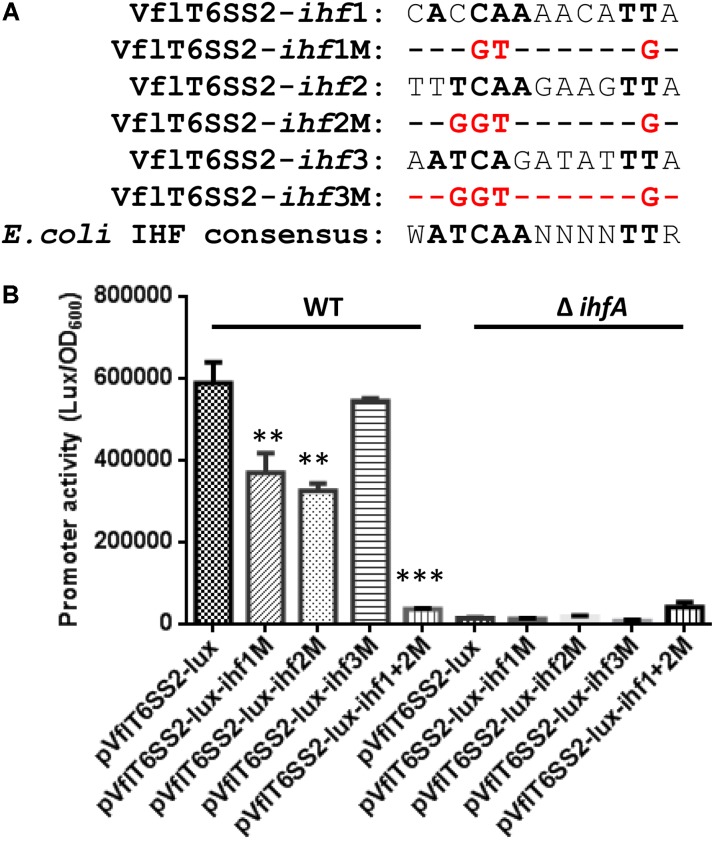
Contributions of the three putative IHF binding sites to the promoter activity of VflT6SS2 major cluster. **(A)** The sequences of the three predicted IHF binding sites within the promoter region of VflT6SS2 major cluster and the mutations that we incorporated into each binding site. The nucleotides identical to the *E. coli* consensus site are in bold. **(B)** Overnight cultures of *V. fluvialis* WT and Δ*ihfA* strains containing either pVflT6SS2-*lux*, pVflT6SS2-*lux*-*ihf*1M, pVflT6SS2-*lux*-*ihf*2M, pVflT6SS2-*lux*-*ihf*3M, or pVflT6SS2-*lux*-*ihf*1+2M reporter fusions were diluted 1:100 in LB medium and 200 μl aliquots were transferred to Opaque-wall 96-well microtiter plates. The plates were incubated at 30°C with shaking for measuring the OD_600_ and light units. Luminescence activity is calculated as light units/OD_600_. The data represent three independent experiments. ^∗∗^Significantly different from pVflT6SS2-*lux* (*t*-test, *P <* 0.05). ^∗∗∗^Significantly different from pVflT6SS2-*lux* (*t*-test, *P <* 0.01).

To further figure out the authentic IHF binding sites among the three predicted ones in the promoter region of VflT6SS2 major cluster, we introduced mutations in each of the three predicted IHF binding sites as depicted in **Figure 9A**. As shown in **Figure 9B**, the luminescence activities of pVflT6SS2-*lux*-*ihf*1M and pVflT6SS2-*lux*-*ihf*2M were apparently decreased compared to its wild-type pVflT6SS2-*lux* but that of pVflT6SS2-*lux*-*ihf*3M did not. These results indicate that IHF might bind to the first and second predicted sites to regulate the expression of VflT6SS2 major cluster. To further confirm these results, we introduced mutations in both of the *ihf*1 and *ihf*2 sites. As shown in **Figure 9B**, the joint mutations of the two sites almost completely abolished the promoter activity of pVflT6SS2-*lux*-*ihf*1+2M. This result confirms that IHF mostly binds to the *ihf*1 and *ihf*2 sites in the promoter of VflT6SS2 major cluster to modulate its expression.

In addition, we checked the sequence conservation of *ihf*1 and *ihf*2 sites at the T6SS major cluster promoters among different *Vibrio* species which share similar T6SS genetic organization. Exactly the same *ihf*1 and *ihf*2 binding sites as in the *V. fluvialis* 85003 were found in another *V. fluvialis* strain ATCC33809 isolated from Bangladesh. In *V. furnissii*, a genetically closest species to *V. fluvialis* among *Vibrionaceae* (Lu et al., 2014), an identical sequence to *V. fluvialis ihf*2 binding site is present. While in *V. cholerae*, no such putative binding sites were identified at the promoter region of T6SS core cluster. These results suggest that the IHF-dependent regulation of the major T6SS cluster may vary in different *Vibrio* species.

## Discussion

The IHF has been implicated in the regulation of over 100 genes with various functions in *E. coli* (Arfin et al., 2000) and *Salmonella enterica* serovar Typhimurium (Mangan et al., 2006). Furthermore, IHF has increasingly been identified as a regulator of virulence gene expression. IHF activates expression of virulence genes *virF*, *virB*, and *icsA* in *Shigella flexneri*, and two main virulence factors *tcpA* and *ctx* in *V. cholerae* (Porter and Dorman, 1997; Stonehouse et al., 2008). IHF is involved in transcriptional regulation of *Brucella abortus virB* operon, which encodes the type IV secretion system (T4SS) (Sieira et al., 2004). In this study, we provide evidences to support that IHF activates the expression of VflT6SS2 and thus antibacterial virulence in *V. fluvialis* by co-regulation of its major cluster and three orphan clusters.

Bioinformatics analysis revealed the presence of putative IHF binding sites at the promoter regions of the three *hcp-vgrG* orphan clusters and the major cluster in *V. fluvialis* VflT6SS2. Deletion of either *ihfA* or *ihfB* resulted in a significant reduction in the expression of both the orphan and the major clusters, suggesting their co-regulation by IHF. Reporter fusion studies, EMSAs and site-directed mutagenesis jointly demonstrated the direct binding and positive transcriptional activation of VflT6SS2 by IHF. Bacterial killing assay clearly showed that lack of IHF impaired the antibacterial virulence of *V. fluvialis* against prey strain *E. coli*, while overexpression of IHF from trans-complemented plasmid not only restored, but also increased the killing activity of *V. fluvialis* predators to a significantly higher level than its WT (**Figure 5**). The co-regulatory mode of the major and orphan clusters by IHF denotes that IHF likely plays a critical role in the control of VflT6SS2 in *V. fluvialis*, where it firstly activates the expression of the major cluster encoding VasH and other structural components, and then together with VasH, it synergistically activates the expression of *hcp*-*vgrG* orphan clusters whose products serve both as the T6SS structural components and effector proteins. In other words, the *hcp*-*vgrG* orphan clusters are under dual control by the global regulator IHF and T6SS specific regulator VasH.

However, it seems that co-regulation of the T6SS major cluster and *hcp*-*vgrG* orphan cluster is not a common feature in *Vibrio* species. The regulation of IHF on *hcp-vgrG* orphan clusters seems more conservative than its regulation on major clusters. The promoters of *hcp* homologues in *V. cholerae*, *V. furnissii* and *V. fluvialis* all contain IHF binding sites which are highly conserved in the locations and sequence compositions (12 bp is identical out of 13-bp binding sequence, **Figure 2**), but the IHF binding sites at the promoters of major clusters show much variation in terms of the number of binding sites and the sequence compositions. EMSA and consensus site mutation analysis (**Figures 8C**, **9**) demonstrated that there are two functional IHF binding sites in the VflT6SS2 major cluster promoter, while sequence comparison analysis revealed lack of or only one less conserved binding site in the corresponding major cluster promoters in *V. cholerae* and *V. furnissii*. So, unlike in the halophilic species, including *V. fluvialis* and *V. furnissii*, IHF may only specifically regulate the *hcp-vgrG* orphan clusters but not T6SS major cluster in the *V. cholerae*. The different regulation mode among different species may reflect or correlate with the distinct survival niches of the species and is worthy of being investigated later.

In addition, our results clearly showed that the three *tssD*2-*tssI*2 orphan clusters of VflT6SS2 display differential expression patterns (**Figure 1**). Combined with our previous data about *tssD*2 mutants (Huang et al., 2017), the results suggest that a moderate *hcp* expression (no less than the level of *tssD*2_b expression) is probably required to keep the function of VflT6SS2 in terms of the Hcp effector secretion and antibacterial virulence activity, and a lower expression (such as similar to *tssD*2_c) cannot maintain the function of VflT6SS2 under general growth conditions. Currently, the mechanism behind the differential expression is still unclear. The promoter sequences of *tssD*2_*a*-*tssI*2_*a* and *tssD*2_*b*-*tssI*2_*b* are highly homology from -228 to -1 bp (98.25% identity) relative to the start codon of the *tssD*2 ORFs, however, low sequence homology exists between -335 and -229 bp, which might be one reason for the differential transcription of *tssD*2_*a* and *tssD*2_*b* through affecting the binding of VasH activator. Sequence alignment analysis of T6SS-associated bacterial enhancer binding proteins (bEBPs) suggests that VasH probably responds to different signals and binds to different DNA sequences (Bernard et al., 2011). However, this hypothesis remains to be examined. VasH has been shown to bind to the promoter region of the *hcp-vgrG* orphan cluster in *V. cholerae*, but its specific binding sequences are not yet determined (Bernard et al., 2011). We do not know whether the two *hcp-vgrG* clusters in *V. cholerae* T6SS were differently expressed as in the *V. fluvialis*, but great sequence divergence does exist in the two *hcp* promoter regions starting from -193 bp relative to the start codon of the ORFs.

The *tssD*2_*c*-*tssI*2_*c* cluster is closely neighbored by three predicted phage integrases on the chromosome, suggesting a possibility of extraneous acquisition. The promoter of *tssD*2_*c*-*tssI*2_*c* cluster is highly heterologous to those of *tssD*2_*a*-*tssI*2_*a* and *tssD*2_*b*-*tssI*2_*b*, with a 225-bp-long sequence interval between the IHF and σ^54^ binding sites rather than a 16-bp interval found in the other two clusters. IHF was found to be necessary for the activation of transcription of some σ^54^ promoters where it acted to assist distant, DNA-bound transcriptional regulators or enhancer-like elements for the initiation of transcription (Freundlich et al., 1992; Engelhorn and Geiselmann, 1998). So, we originally inferred that the long sequence interval between the IHF and σ^54^ binding sites in *tssD*2_*c* promoter may somehow account for its low transcriptional activity, but experimental analysis of *tssD*2_*c* promoter with artificially shortened interval revealed that the interval sequence is not the reason, instead, it contains a probable *cis*-acting element required for maintaining its basal transcriptional activity.

The physiological significance of containing multiple copies of *hcp-vgrG* genes in T6SS system in *V. fluvialis*, as seen in other bacteria, is still unclear, and the same question for their differential expressions. To some extent, this may represent an alternative regulatory mechanism which selectively expresses *hcp-vgrG* pairs at certain conditions, allowing the bacteria to produce distinct Hcp/VgrG structures or forming different cocktails of Hcp/VgrG structures (Bernard et al., 2011). Hcp is not only the structural component forming 600-nm-long homohexameric inter tube through which the toxin effectors was loaded and secreted (Journet and Cascales, 2016), but also serves as an important chaperone for T6SS effectors by being secreted together with them to prevent their degradation (Silverman et al., 2013). We speculate that the chaperone function of Hcp may be benefited from the multicopy and colocation with effector VgrGs within the different clusters.

**FIGURE 10:**
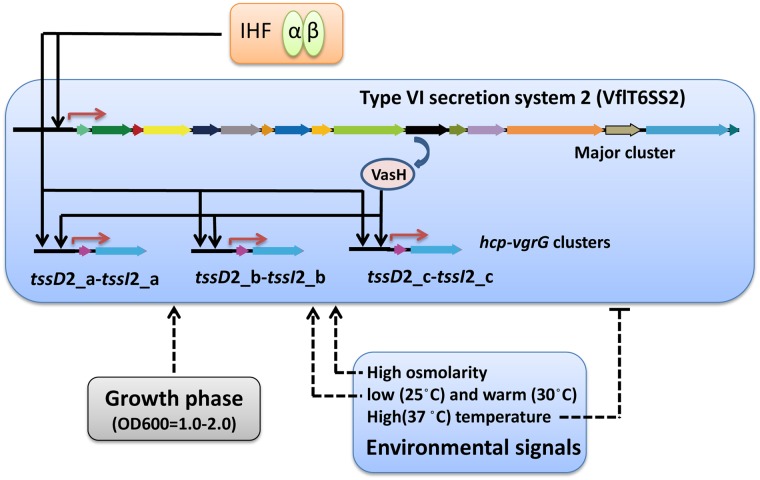
Schematic representation of the regulation of *V. fluvialis* VflT6SS2. Activation is designated by arrow-headed lines while inhibition is indicated by bar-headed lines. Solid lines in the VflT6SS2 bubble represent the direct binding to the promoter by the regulators. Dashed lines that do not enter the VflT6SS2 bubble represent regulation through unknown mechanisms. Red arrows denote transcriptional start sites.

Taken together, we demonstrated here that functional expression of VflT6SS2 in *V. fluvialis* was positively regulated by the global regulator IHF. Current results add new information to the highly complex regulatory circuitry controlling the expression of T6SS and further broaden our knowledge of T6SS regulation. In **Figure 10**, we propose a model for the expression and regulation of VflT6SS2 in *V. fluvialis*, including transcriptional regulators and environmental signals. Specifically, IHF positively co-regulates the VflT6SS2 major cluster and *hcp-vgrG* orphan clusters, and the orphan clusters undergo dual regulation of IHF and VasH. Environmental conditions, such as growth stage at OD_600_ 1.0-2.0, high osmolality, and low (25°C) or warm (30°C) temperature facilitate while high temperature (37°C) represses VflT6SS2 expression.

## Author Contributions

WL and BK conceived and designed the experiments. JP, MZ, YH, and XL performed the experiments. WL, JP, JL, and ZR analyzed the data and discussed the results. WL and JP wrote the paper.

## Conflict of Interest Statement

The authors declare that the research was conducted in the absence of any commercial or financial relationships that could be construed as a potential conflict of interest.

## Footnotes

1 http://www.prodoric.de/vfp/

## Abbreviations

Δ*ihfA*: isogenic *ihfA* mutant
Δ*ihfB*: isogenic *ihfB* mutant
CFU: colony-forming unit
EMSA: electrophoretic mobility shift assay
Hcp: hemolysin-coregulated protein
IHF: integration host factor
LB: Luria-Bertani broth
OD_600_: optical density at 600 nm
ORF: open reading frame
PCR: polymerase chain reaction
qRT-PCR: quantitative reverse transcription PCR
T6SS: type VI secretion system
WT: wild-type
